# The Adsorption Behaviors and Mechanisms of Humic Substances by Thermally Oxidized Graphitic Carbon Nitride

**DOI:** 10.3390/toxics11040369

**Published:** 2023-04-12

**Authors:** Hongxin Li, Jianlong Wang, Dongbei Yue, Jianchao Wang, Chu Tang, Lingyue Zhang

**Affiliations:** 1 School of Environment and Energy Engineering, Beijing University of Civil Engineering and Architecture, Beijing 100044, China; 2 School of Environment, Tsinghua University, Beijing 100084, China; 3 School of Chemical and Environmental Engineering, China University of Mining and Technology (Beijing), Beijing 100083, China; 4 School of Department of Civil Engineering, The University of Hong Kong, Pokfulam 999077, Hong Kong SAR, China

**Keywords:** humic substances, adsorption removal, π-π interactions, electrostatic interactions

## Abstract

Thermal oxidation is efficient for enhancing the photocatalysis performance of graphitic carbon nitride (g-C_3_N_4_), while its effect on adsorption performance has not been fully studied, which is crucial to the application of g-C_3_N_4_ as adsorbents and photocatalysts. In this study, thermal oxidation was used to prepare sheet-like g-C_3_N_4_ (TCN), and its application for adsorption of humic acids (HA) and fulvic acids (FA) was evaluated. The results showed that thermal oxidation clearly affected the properties of TCN. After thermal oxidation, the adsorption performance of TCN was enhanced significantly, and the adsorption amount of HA increased from 63.23 (the bulk g-C_3_N_4_) to 145.35 mg/g [TCN prepared at 600 °C (TCN-600)]. Based on fitting results using the Sips model, the maximum adsorption amounts of TCN-600 for HA and FA were 327.88 and 213.58 mg/g, respectively. The adsorption for HA and FA was markedly affected by pH, alkaline, and alkaline earth metals due to electrostatic interactions. The major adsorption mechanisms included electrostatic interactions, π-π interactions, hydrogen bonding, along with a special pH-dependent conformation (for HA). These findings implied that TCN prepared from environmental-friendly thermal oxidation showed promising prospects for humic substances (HSs) adsorption in natural water and wastewater.

## 1. Introduction

Humic substances (HSs), including humic acid (HA) and fulvic acid (FA), are commonly found in various water bodies, such as surface water, underground water, wastewater, and landfill leachate [1]. In chemical compositions, HSs are mixtures of aliphatic and aromatic organic compounds and have abundant oxygen-containing groups, such as phenolic alcohol (-OH), methoxycarbonyl (-C-O), and carboxylic acid (-COOH) groups [2]. Naturally occurring HSs (5–20 mg/L) are not hazardous to human health, while due to abundant chemical functional groups, HSs have strong bonding affinity to heavy metals, pesticides, microplastics, and antibiotics, resulting in serious environmental pollution and health risks [3]. Particularly in chlorination disinfection, HSs can react with chlorine, generating highly carcinogenic disinfection by-products, such as trihalomethanes and haloacetic acids, which are highly toxic to humans and cause serious crisis for the sustainable supply of drinking water [4]. Recently, HSs have been regarded as ultraviolet quenching substances (UVQS), which can lower the UV transmittance and strongly decrease the efficacy of UV disinfection [5]; at the same time, as an emerging environmental problem, UVQS has attracted increasing attention from researchers and engineers. In addition, the accumulation of HSs in water bodies can cause serious organoleptic issues, such as unpleasant colors (yellowish to black) and undesirable odors. Consequently, the removal of HSs from water bodies is of important significance to control the environmental problems induced by HSs.

Several methods for HSs elimination from the aqueous solutions have been developed, such as electrocoagulation, advanced oxidation processes, membrane filtration, biological treatment, and adsorption [6]. Of these, adsorption is a widely used method due to its high removal efficiency, convenient operation, low investment, and reusability. In previous studies, several adsorbents, such as fly ash, clay minerals, and activated carbon, have been used for adsorption of HSs [7,8]. However, these adsorbents were not efficient for adsorption of HSs due to the inherent structural properties. For example, the micropore of activated carbon was not available for HSs adsorption due to the strong size exclusion effect. In addition, nanosized adsorbents, such as zero-valent iron [9] and layered double hydroxides-Fe_3_O_4_ nano-composites [10], were used for adsorption of HSs due to their large surface area. However, the nanosized adsorbents suffer from high cost and aggregation, limiting the practical application of these adsorbents [11]. Moreover, most of the nanosized adsorbents use toxic raw materials in the synthesis process, and thus the post-consumer counterparts have high eco-toxicity [8]. Therefore, the fabrication of cost-efficient and environmentally friendly adsorbents for adsorption of HSs has attracted more and more attention [12].

Recently, graphitic carbon nitride (g-C_3_N_4_), an emerging material, has been widely used for water splitting and photodegradation of organic pollutants due to its abundant precursors, simple synthesis, thermal/chemical stability, and environmental friendliness [13]. For example, the efficient mineralization of trichloroethylene using S-scheme heterojunctions of g-C_3_N_4_ was reported [14]. In the literature focusing on g-C_3_N_4_, most of the studies pay more attention to the photocatalysis performance, while there are few studies using g-C_3_N_4_ as adsorbents [15]. The current studies using g-C_3_N_4_-based adsorbents mainly focused on the adsorption of heavy metals, since pyridine-like nitrogen can efficiently capture cations via ligands due to the rich electron lone pairs. For example, Xiao et al. and Zhu et al. studied the adsorption capacity for lead (II) onto g-C_3_N_4_, which was synthesized from melamine via the pyrolysis method [16,17]. Tan et al. and Guo et al. reported the adsorption of cadmium (II) on virgin g-C_3_N_4_ and magnetic g-C_3_N_4_ [18,19]. However, there are few studies about g-C_3_N_4_ focusing on the adsorption of organic matters. Zhu et al. and Santoso et al. [20] reported the adsorption of methylene blue on g-C_3_N_4_. Yan et al. [21] reported that g-C_3_N_4_ showed great adsorption performance for perfluorooctane sulfonate. More recently, we revealed that g-C_3_N_4_ showed promise for adsorption of HSs, but the small specific surface area of g-C_3_N_4_ strongly restricts its application [22]. This is because the g-C_3_N_4_ materials are prepared via the pyrolysis method using precursors with earth-abundant carbon and nitrogen elements. Although the theoretical surface area for ideal monolayer g-C_3_N_4_ can be as high as 2500 m^2^/g, the g-C_3_N_4_ materials prepared using pyrolysis exhibited a stacked structure, particularly for g-C_3_N_4_ derived from thiourea, melamine, and cyanamide [23]. As reported by Wang et al. [22], the specific surface area of bulk g-C_3_N_4_ derived from melamine, thiourea, and cyanamide was 10.35, 11.24, and 9.32 m^2^/g, respectively, and the bulk g-C_3_N_4_ showed a very low performance for HSs adsorption. This makes the g-C_3_N_4_ materials with large surface area or with nanoscale structures highly desired for application as adsorbents.

Some methods, such as soft and hard templating methods, have been reported to prepare porous g-C_3_N_4_ with a high specific surface area. Yan [24] reported that using Pluronic P123 as a soft template, the worm-like porous g-C_3_N_4_ with a high surface area and great photocatalytic activity was prepared. Nevertheless, the soft template has undesirable residues of template polymers, which affects the performance of the materials, and the hard templating consumes hazardous hydrogen fluoride [14]. In this regard, a simple, efficient, and environmentally friendly thermal oxidation was proposed to prepare g-C_3_N_4_ with a high surface area [25]. This method remarkably affects the surface area and porosity of g-C_3_N_4_, leading to an improved photocatalysis. For instance, thermal oxidation was used to prepare porous g-C_3_N_4_ nanosheets, and the prepared g-C_3_N_4_ showed an enhanced visible light photocatalytic activity, and the radiative lifetime of charge carriers (τ_1_ and τ_2_) increased from 4.13 and 26.23 ns to 5.36 and 36.57 ns after thermal exfoliation, respectively [26]. The mechanisms are that thermal oxidation can destroy the layer structures of g-C_3_N_4_ bonded by hydrogen bonding and Van der Waals force. However, the effect of thermal oxidation on the adsorption performance of g-C_3_N_4_ has not been fully investigated, particularly for the effect of porosity and surface chemistry of g-C_3_N_4_ on its adsorption performance. Particularly, given that melamine was a most-used precursor of g-C_3_N_4_, while the effect of thermal oxidation on its adsorption performance for organic pollutants has not been studied. On the other hand, in heterogeneous photocatalysis using g-C_3_N_4_, the organics are adsorbed on the surface of g-C_3_N_4_ and then photodegraded, since free radicals cannot leave the surface of g-C_3_N_4_ due to a short half-life [27]. The adsorption of g-C_3_N_4_ for organics is a critical process, while this critical process is rarely elucidated, and only some reports studied the adsorption of methylene blue, a typical pollutant during photocatalysis [20]. Consequently, studies on the adsorption performance of g-C_3_N_4_ derived from melamine after thermal oxidation is important for the application of this emerging material as an adsorbent and photocatalyst.

Therefore, this study investigated the application of melamine-derived g-C_3_N_4_ after thermal oxidation for adsorption of HSs. First, the changes in the textural properties of g-C_3_N_4_ after thermal oxidation (TCN) were studied. Second, the adsorption kinetics and isotherms for HA and FA to TCN were investigated. The effect of contact time, initial pH, initial concentration, metal ions, and temperature on adsorption of HSs was further studied. Third, the adsorption mechanisms of HSs on TCN and the changes in the surface properties of TCN after adsorption were elucidated.

## 2. Materials and Methods

### 2.1. Preparation of Adsorbents and HS Materials

HSs materials were obtained from the Macklin Co., Ltd. (Shanghai, China). The precursor of g-C_3_N_4_, analytical grade melamine, was purchased from Sinopharm Chemical Reagent Co., Ltd., (Shanghai, China). The leachate nanofiltration concentrate was collected from the treatment plant located in Beijing, China, which was filtered through a 0.45 μm membrane and stored at 4 °C. The chemical properties of landfill leachate concentrate were: pH = 9.06, dissolved organic carbon (DOC) = 1122.92 mg C/L, conductivity = 26,487.83 μs/cm, and UV_254_ = 36.00 (/cm). The bulk g-C_3_N_4_ was synthesized via the pyrolysis of precursor at 500 °C for 2 h in air. After milling, the bulk g-C_3_N_4_ was then subjected to thermal oxidation. The thermal oxidation was conducted at 500, 550, and 600 °C for 3 h in air, and the resultant g-C_3_N_4_ samples were denoted as TCN-500, TCN-550, and TCN-600, respectively.

### 2.2. Characterization of Adsorbents

X-ray diffraction (XRD, Zürich, Switzerland) was used to characterize the crystalline structure of the adsorbents. The chemical composition of the adsorbents was analyzed using Fourier transforms infrared spectra spectroscopy (FT-IR, Bartlett, IL USA) and X-ray photoelectron spectroscopy (XPS, Hopkinton, MA, USA). The morphology of the adsorbents was recorded with field emission scanning electron microscopy (SEM, Hitachi, Tokyo, Japan). The Brunauer–Emmett–Teller (BET) surface area and pore-size distribution of the adsorbents were determined with Quanta Chrome ASAP 2460 analyzer (Micromeritics, Norcross, GA, USA). The zeta potential of the adsorbents was measured with an instrument at 25 °C with suspensions containing 1 g/L of solids (Delsa Nano C, Beckman Coulter, Brea, CA, USA). The pH of the solutions was adjusted using 0.1 M NaOH and HCl.

### 2.3. Adsorption Procedures

For each batch adsorption experiment, 0.04 g of g-C_3_N_4_ was added to 100 mL of HA or FA solution, and the adsorption was performed at 298.5 K in the dark using an instrument (Figure S1). The adsorption of HA or FA was systematically investigated according to the following parameters: initial concentration of HSs (25–200 mg/L), contact time (0–240 min), initial pH (2.0–10.0), ionic strength (I, NaCl, 0–0.07 M), temperature (298.15–318.15 K), alkali metals (K^+^, 0–0.07 M), and alkali earth metals (Ca^2+^, Mg^2+^, 0–1.05 mM) (Table S1). HSs concentration was measured using a UV-visible spectrometer (UV-1800, Shimadzu, Kyoto, Japan) at a wavelength of 254 nm. The experiments were conducted in duplicate at least, and the average values were reported. At equilibrium, the removal rate and adsorption capacity were calculated as the following equations:

(1) $$R=\frac{C_{0}-C_{e}}{C_{0}}\times 100\%$$

(2) $$q_{e}=\frac{\left(C_{0}-C_{e}\right)V}{m}$$

where R (%) and $q_{e}$ (mg/g) are the removal rate and adsorption capacity, respectively. m (g) is the mass of absorbents. $C_{0}$ is the initial concentration (mg/L). $C_{e}$ is the equilibrium concentration (mg/L). V (L) is the volume of HSs solution.

The kinetics data were simulated with the pseudo-first-order (PFO) model, the pseudo-second-order (PSO) model, and the Elovich kinetic model according to the following equations, respectively:

(3) $$q_{t}=q_{e}\left(1-e^{-k_{1}t}\right)$$

(4) $$q_{t}=\frac{q_{e}^{2}k_{2}t}{\left(1+q_{e}k_{2}t\right)}$$

(5) $$q_{t}=\frac{1}{\beta }\ln\left(1+\alpha \beta t\right)$$

where $q_{e}$ (mg/g) and $q_{t}$ (mg/g) are the equilibrium and adsorption capacities at time t, respectively. $k_{1}$ (g/mg/min) and $k_{2}$ (g/mg/min) are the rate constants of the PFO and PSO models, respectively. α is the initial adsorption rate (mg/g/min). β is the desorption constant (g/mg) and t (min) is the contact time in the Elovich equation.

The intraparticle diffusion model was used to study the intraparticle diffusion process, as described by the following equation:

(6) $$q_{t}=k_{id}t^{1/2}+C$$

where $k_{id}$ [(mg/g/min^1/2^)] and C (mg/g) are the rate constant and the boundary layer thickness constant, respectively.

The Freundlich, Langmuir, Sips, and Temkin models were used to describe the adsorption isotherms as follows:

(7) $$q_{e}=k_{f}C_{0}^{\frac{1}{n}}$$

(8) $$\frac{C_{e}}{q_{e}}=\frac{C_{e}}{q_{m}}+\frac{1}{k_{l}q_{m}}$$

(9) qe=qmksCe1n1+ksCe1n

(10) $$q_{e}=\frac{RT}{bY}lnA_{T}C_{e}$$

where $k_{f}$ [(mg/g) (L/mg)^1/n^], $k_{l}$ (L/mg), and $k_{s}$ (L/mg) are the constants for the Langmuir, Freundlich, and Sips models, respectively. R is 8.314 J/mol·K. T (K) is temperature. $A_{T}$ (L/g) and $b_{T}$ are the constants of Temkin. $C_{e}$ (mg/L) is the equilibrium concentration of HSs. $q_{m}$ (mg/g) is the maximum adsorption capacity. 1/n is the heterogeneity factor.

The thermodynamic parameters, including the Gibbs free energy ($\Delta G^{0}$, kJ/M), enthalpy ($\Delta H^{0}$, kJ/M), and entropy ($\Delta S^{0}$, J/M/K) were calculated as follows:

(11) $$ln\frac{1}{C_{e}}=lnk_{0}-\frac{\Delta H^{0}}{RT}$$

(12) $$\Delta G^{0}=-nRT$$

(13) $$\Delta S^{0}=\frac{\Delta H^{0}-\Delta G^{0}}{T}$$

where T is temperature (K). $K_{0}$ is the thermodynamic constant. The values of $\Delta H^{0}$ and $\Delta S^{0}$ were calculated from the slope and intercept of the linear regression of $lnK_{0}$ versus $T^{-1}$.

### 2.4. Adsorption Mechanisms

The adsorption mechanisms were ascertained using dynamic light scattering (DLS), XPS, nitrogen adsorption–desorption isotherms, along with fluorescence excitation-emission matrix (EEM). DLS was utilized to evaluate the changes in the conformation of HSs with respect to pH using a Nanoparticle Size Analyzer (Delsa Nano C, Indianapolis, IN, USA) for a special adsorption mechanism. XPS was used to determine the differences in the binding energy before and after adsorption. The porosity of the adsorbents after HSs adsorption was investigated using nitrogen adsorption–desorption isotherms. EEM was recorded with a spectrofluorometer (F-7000, Hitachi) to investigate the changes in the fluorescence features of HSs before and after adsorption on TCN-600.

## 3. Results and Discussion

### 3.1. Characterization of Adsorbents

**Figure 1:**
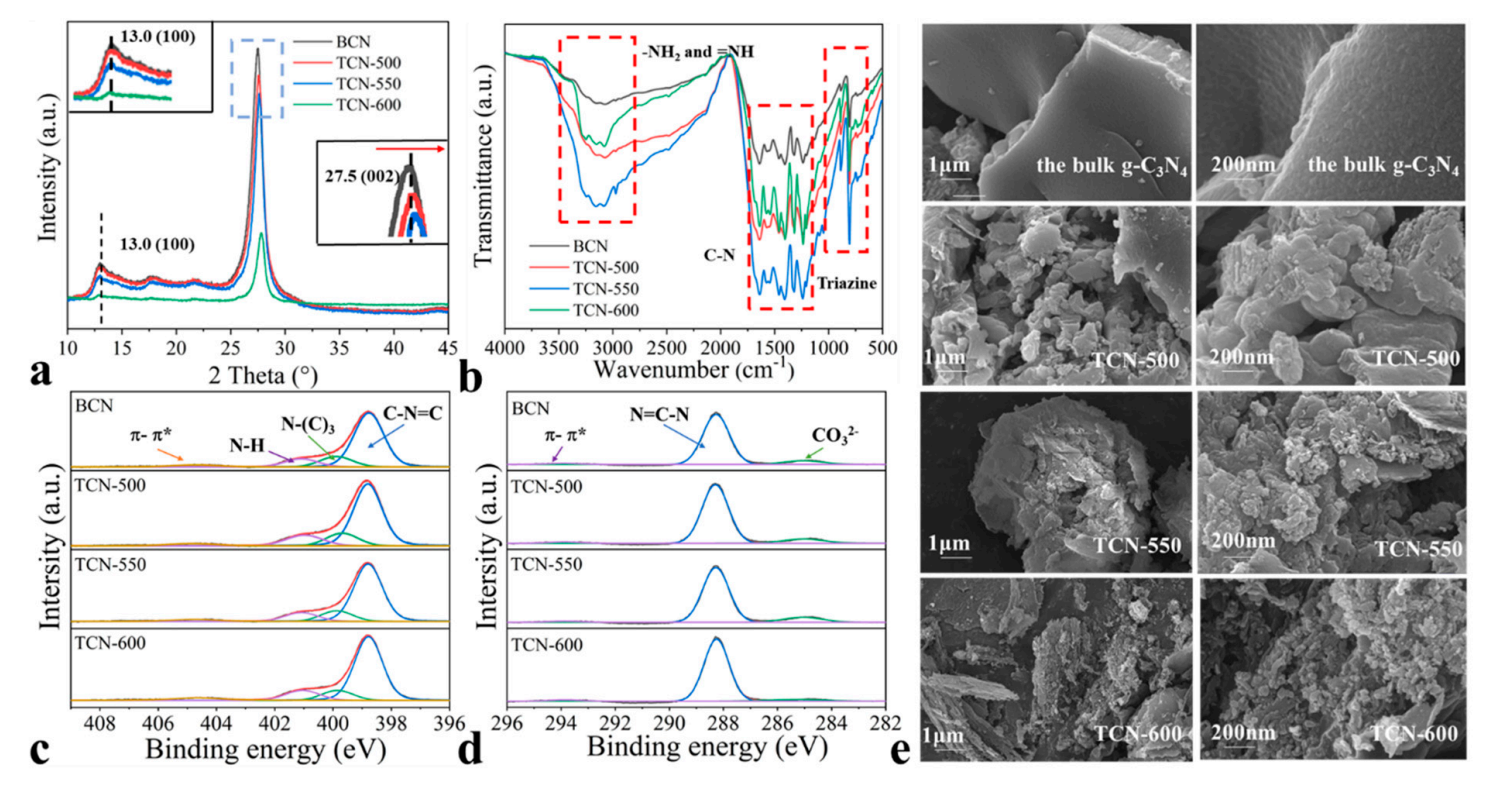
(**a**) The XRD patterns, (**b**) FT-IR spectra, (**c**) N 1s core region, (**d**) C 1s core region, and (**e**) SEM images of the bulk g-C_3_N_4_ and TCN samples.

The crystal structure of the bulk g-C_3_N_4_ and TCN samples was characterized by XRD patterns. As shown in Figure 1a, there were two diffraction peaks at approximately 27.5° and 13.0°. The stronger peak at 27.5° was ascribed to the interlayer superposition of reflections in graphite, corresponding to the 002 crystal face of g-C_3_N_4_ [28]. The 002 peak was consistent with the known accumulation peak of aromatic systems. With the increasing of thermal oxidation temperature, the intensity of the 002 diffraction peak of TCN (27.55°) samples was higher than that of bulk g-C_3_N_4_ (27.35°). This indicated that the crystallinity of TCN samples increased. The reflection peak at 13.0° was attributed to the interlayer tri-s-triazine units stacking, which was indexed as (100) [28].

The functional groups of the bulk g-C_3_N_4_ and TCN samples were studied using FT-IR. As shown in Figure 1b, there were no differences in the FT-IR of the adsorbents. There were three distinct groups of bands, which were consistent with that of the typical g-C_3_N_4_ materials, in agreement with the previous reports [29]. The sharp peak at 800 cm^−1^ was related to the rings of triazine or heptazine, the absorption bands in the range of 1100–1600 cm^−1^ were the asymmetric stretching of C-N and C=N heterocycles, and the broad bands in the range of 3000–3500 cm^−1^ were the stretching vibrations of the -N-H or -N-H_2_ groups derived from the uncondensed amino groups [30].

The surface compositions and chemical states of the adsorbents were investigated using XPS (Figure 1c,d, Tables S2 and S3). As shown in Figure 1c, there were four peaks at binding energies of 398.8, 399.6, 400.9, and 404.5 eV, respectively. The peaks of 398.8 and 399.6 eV were attributed to the sp^2^-hybridized nitrogen bonded to carbon (C-N=C) and bridging N atoms (N-(C)_3_), respectively. The peaks of 400.9 and 404.5 eV related to the primary or secondary amino groups (-NH_2_ or =NH) and charging effect, respectively [31]. As shown in Figure 1d, there were three peaks at 284.8, 288.3, and 293.8 eV in the C 1s region; the first peak was attributed to adventitious carbon, the second was the sp^2^-hybridized carbon, and the third peak was π-π* excitation [32].

As shown in Figure 1e, the morphological structure and interlayer stacking of the adsorbents were observed. The bulk g-C_3_N_4_ had a nonporous structure, whereas the TCN samples exhibited porous structure. The TCN-500 samples displayed a layered structure composed of large lumps with a few pores and exhibited a reduced thickness with more pores. When the thermal temperature was increased to 600 °C, the TCN-600 samples showed a thinner sheet-like structure with abundant pores. As the temperature of thermal oxidation increased, the thickness of TCN decreased and possessed more abundant pores, indicating that the thermal oxidation induced morphological changes, in agreement with the previous studies [25]. As previously reported [33], the energy of van der Waals forces and of hydrogen bonding in the interlayers of g-C_3_N_4_ are fragile and can be broken after thermal oxidation. The formation of pores was ascribed to the decomposition of g-C_3_N_4_ into gaseous products [25]. The yield of the adsorbents decreased with increasing thermal temperature, which was consistent with the above findings.

**Figure 2:**
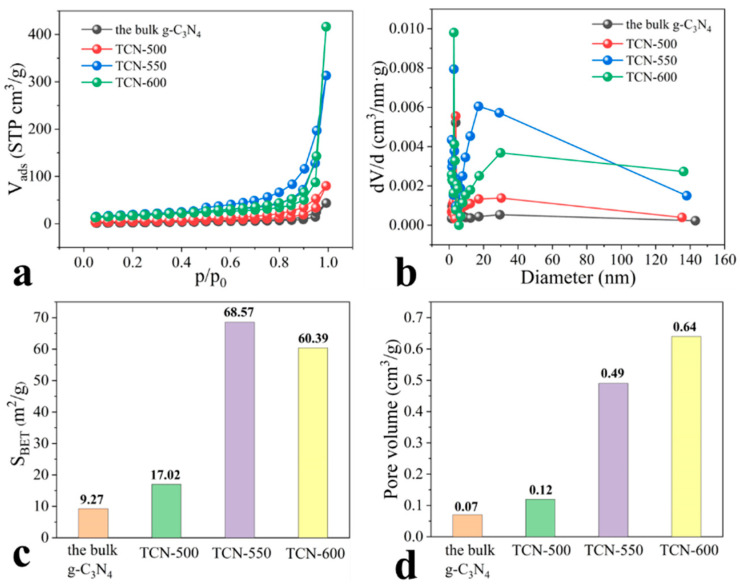
(**a**) The nitrogen adsorption–desorption isotherms, (**b**) pore-size distribution, (**c**) surface areas, and (**d**) pore volume of the bulk g-C_3_N_4_ and TCN.

The nitrogen adsorption–desorption and pore-size distribution curves of the adsorbents are shown in Figure 2. The surface area of the TCN samples increased after thermal oxidation. Notably, the surface area of TCN-550 and TCN-600 was 68.57 and 60.39 m^2^/g, respectively, which were much higher than those of the bulk g-C_3_N_4_. In addition, the pore volume of the TCN samples steadily increased with the increased thermal oxidation temperature, and that of TCN-600 reached 0.64 cm^3^/g. All isotherms exhibited a H3-type hysteresis loop, suggesting the formation of slit-shaped pores [34]. In contrast to the bulk g-C_3_N_4_, the hysteresis loop of TCN samples shifted to lower pressure, and the area of the hysteresis loop grew with the increased thermal temperature, indicating the formation of a relatively large mesopore [35]. The above findings can be explained by the reduced layer thickness and size of the TCN samples, which were consistent with the results of morphological features. This was consistent with the surface area and pore-size distribution for the bulk g-C_3_N_4_ and TCN samples. These variations in the porosity of the TCN samples would affect their adsorption performance for HSs. The increased surface area and pore volume provided more active adsorption sites for HSs adsorption. For macromolecular HSs, however, the pore size also affects adsorption, besides the specific surface area.

### 3.2. Comparison of HA Adsorption Using Different Adsorbents

The adsorption capacities and removal rates of HA (as an example) by the bulk g-C_3_N_4_ and TCN are shown in Figure S2. It was found that TCN adsorbed greater amounts of HA with higher removal rates than the bulk g-C_3_N_4_. The amount of HA adsorbed by TCN-600 was 145.35 mg/g (more than twice that of the bulk g-C_3_N_4_). This was consistent with the changes in surface area and pore volume, suggesting that TCN-600 had greater potential for HSs removal because its large surface area and pore volume provide more active sites. Moreover, it was found that, although the surface area of TCN-550 was similar to that of TCN-600, the amount of HA adsorbed to TCN-600 was greater than that to TCN-550. This was due to the higher pore volume of TCN-600.

### 3.3. Adsorption Kinetics

**Figure 3:**
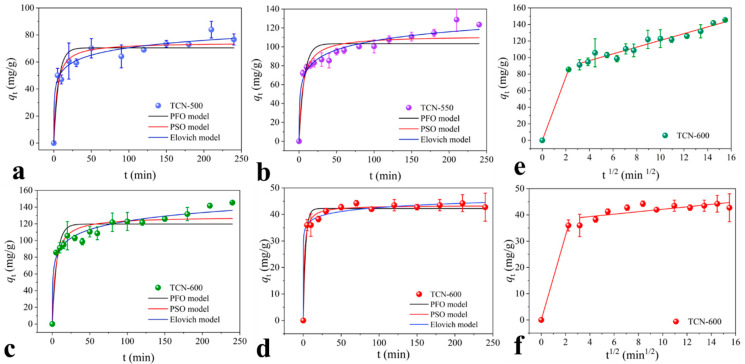
(**a**–**c**) The adsorption kinetics of HA on TCN (C_HSs_ = 100 mg/L, pH = 3.0, T = 298.5 K, C_TCN_ = 0.4 g/L, and I = 0.01 M); (**d**) The adsorption kinetics of FA on TCN-600 (C_HSs_ = 50 mg/L, pH = 3.0, T = 298.5 K, C_TCN-600_ = 0.4 g/L, and I = 0.01 M); (**e**,**f**) The intraparticle diffusion of HA and FA on TCN-600 [C_HSs_ = 100 (HA) or 50 (FA) mg/L, pH = 3.0, T = 298.5 K, C_TCN-600_ = 0.4 g/L, and I = 0.01 M].

The adsorption kinetics of HA and FA on the bulk g-C_3_N_4_ and TCN are shown in Figure 3 and Figure S3. It was observed that the adsorption amounts of HA and FA increased until reaching equilibrium for both the bulk g-C_3_N_4_ and TCN. Within the initial 30 min, the adsorption amounts of HA and FA showed a significant increase. This phenomenon was attributed to abundant active sites within the initial stage. The active sites became more completely bound and eventually saturated with the contact time increased, resulting in the decreased adsorption rate. In terms of HA adsorption, TCN-600 showed the greatest adsorption amount, which was consistent with the results of adsorption capacities. In addition, TCN-600 exhibited greater adsorption capacity for HA than for FA, indicating a higher adsorption affinity towards HA.

For the adsorption of HA and FA on the bulk g-C_3_N_4_ and TCN, the adsorption kinetic data were fitted according to the PFO, PSO, and Elovich models (Figure 3 and Table S4). It was found that the correlation coefficient ($R^{2}$) of the PSO-fitted data was higher than that of the PFO-fitted data, suggesting that the adsorption of HA and FA is related to chemisorption [7]. Among these models, the Elovich model showed the best fitting, as indicated by the highest $R^{2}$ value. The Elovich model is often used to describe chemisorption on heterogeneous adsorbing surfaces [36]. Collectively, it was speculated that HSs adsorption is related to chemisorption.

As shown in Figure 3e,f, the diffusion process for adsorption of HSs to TCN-600 was ascertained based on the intraparticle diffusion model. It was found that the correlation curves of $q_{t}$ and $t^{1/2}$ were multilinear, indicating that boundary layer diffusion and interior adsorption were involved in the adsorption of HSs to TCN-600. In the first stage, HA and FA molecules migrated from the solution to the external surface of TCN-600 with a high mass transfer rate. However, in the second stage, the steric effect slowed the mass transfer rate of macromolecular HA and FA, resulting in less intraparticle diffusion. A similar phenomenon was reported for adsorption of HSs on carbon nanotubes [37].

### 3.4. Effect of pH on Adsorption of HSs

The effect of pH on the adsorption of HA and FA on TCN-600 was investigated at pH 2.0–10.0. The adsorption amounts of HA and FA decreased rapidly as pH increased (Figure S4). This was consistent with that of a previous study about adsorption of HSs on activated carbon [38]. When pH was increased from 2.0 to 10.0, the adsorption amounts of HA and FA decreased from 113.55 to 9.08 mg/g and from 56.99 to 15.75 mg/g, respectively. Thus, the acidic condition apparently favored adsorption of HSs to TCN-600. This indicated that electrostatic interactions played an essential role in HSs adsorption. Moreover, the zeta potential of TCN-600 was measured as a function of pH (Figure S5). It was found that the surface of TCN-600 was positive when pH of the solution was below 3.0. The effect of pH on adsorption of HSs can be explained from two points. On the one hand, the surface of TCN contained abundant amino groups, which were protonated or deprotonated, resulting in negatively or positively charged surfaces. The carboxylic and phenolic groups of HSs could be protonated or deprotonated [39]. When pH was increased, the surface charge of TCN-600 became more negative, rendering TCN-600 more repulsive towards the negatively charged HSs. Accordingly, the decreased adsorption with pH was attributed to enhanced electrostatic repulsion. On the other hand, by contrast to FA adsorption, HA adsorption was more dependent on pH. This was possibly due to the pH-dependent conformation (a non-interaction adsorption mechanism), and HA could become more compact with a higher adsorption affinity at low pH values [40]. In order to verify this, DLS analysis of HA and FA with respect to pH was conducted (Figure S6), and the DLS results indicated that as pH increased, the size of HA increased due to the decoiling of macromolecular HA, in agreement with the studies reported by Wells and Stretz [41]. By contrast, the size of FA kept stable as pH increased. Accordingly, the adsorption of HA was affected by pH via electrostatic interactions and the special pH-dependent conformation.

### 3.5. Effect of the Common Ions on Adsorption of HSs

**Figure 4:**
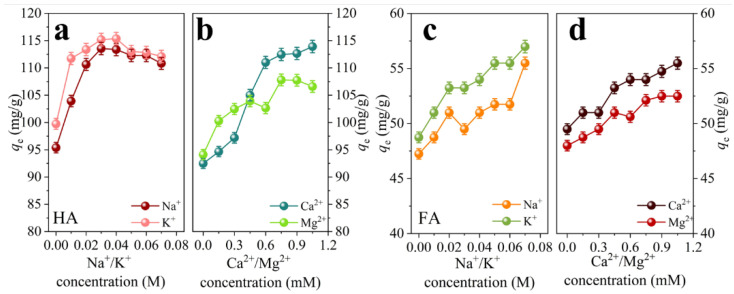
The effects of (**a**,**c**) alkali metals and (**b**,**d**) alkali earth metals [C_HSs_ = 50 mg/L, pH = 3.0, T = 298.5 K, C_TCN-600_ = 0.4 g/L, I = 0.01 M (for K^+^, Ca^2+^, and Mg^2+^)] on the adsorption of HA and FA to TCN-600.

The alkali metals (Na^+^ and K^+^) and alkali earth metals (Ca^2+^ and Mg^2+^) are ubiquitous in water. Thus, the effect of these ions on the adsorption of HA and FA to TCN-600 was investigated. When the concentration of the alkali and alkali earth metals was increased, the adsorption amounts of HA and FA increased significantly, as shown in Figure 4. This further elucidated electrostatic interactions as a primary mechanism for adsorption of HSs to TCN-600, consistent with the effect of pH. Thus, the increase in adsorption of HSs with the increase of alkali and alkali earth metals was due to the resulted compressed electrical double layer, reducing electrostatic repulsion between HSs and TCN [42]. As the concentration of ionic increased, the thickness of the electrical double layer was compressed, causing HSs to adhere to TCN-600 more closely. Moreover, the precipitation or aggregation of HSs at high ionic strengths could counteract electrostatic repulsion. At higher ionic strengths, HA formed a smaller configuration, thus occupying fewer adsorption sites and leading to a greater adsorption capacity to TCN-600 [43]. As shown in Figure 4b,d, the alkali earth metals (Ca^2+^ and Mg^2+^) were favorable for the adsorption of HA and FA to TCN-600. This can be attributed to the role of Ca^2+^ and Mg^2+^ in neutralizing the negative charges, reducing electrostatic repulsions between HSs and TCN-600. Moreover, the effect of Ca^2+^ on the adsorption HA and FA to TCN-600 was greater than that of Mg^2+^ due to the differences in ionic size. This was consistent with the adsorption of HA by nano-amorphous calcium phosphate [44].

### 3.6. Adsorption Isotherms and Thermodynamic Analysis

**Figure 5:**
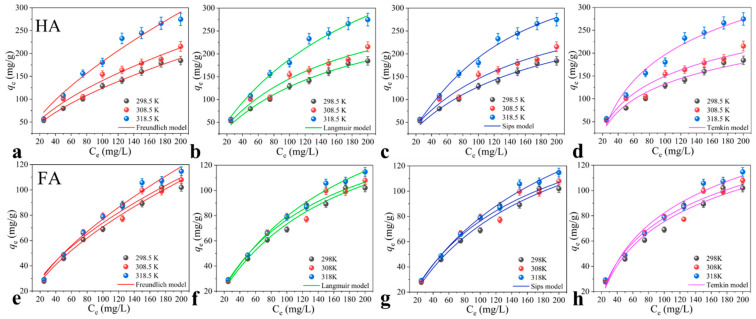
The adsorption isotherms of (**a**–**d**) HA and (**e**–**h**) FA to TCN-600 (C_HSs_ = 25–200 mg/L, pH = 3.0, C_TCN-600_ = 0.4 g/L, and I = 0.01 M, T = 298.5, 308.5, and 318.5 K).

Temperature was an important factor affecting adsorption of HSs, and thus the adsorption experiments were carried out at different temperatures. As shown in Figure 5, at the same temperature, the adsorption amounts of HA and FA increased with increasing initial concentration due to the concentration gradient. Apparently, the temperature had no noticeable effect on FA adsorption. By contrast, the adsorption amounts of HA increased with the increasing of temperature. The isotherm data were fitted using the two-parameter models (Freundlich, Langmuir, and Temkin) and a single three-parameter model (Sips). As shown in Table S5, all four models well described the adsorption isotherms of HSs to TCN, and the correlation coefficients $R^{2}$ were greater than 0.950. However, the Langmuir and Sips models fitted better compared to the other models. The Langmuir model is generally used to describe the uniform distribution of surface-active centers [45]. The Sips model combines the Langmuir and Freundlich isotherm models and is used to analyze the heterogeneous adsorption systems and monolayer adsorption characteristics at high concentration of adsorbate [46]. In our case, the $k_{s}$ and 1/n values of the Sips model were close to zero and one, respectively, suggesting that the adsorption of HA and FA to TCN-600 was heterogeneous, and the surface of TCN-600 possessed heterogeneous adsorption sites for adsorption of HSs.

In addition, according to the Sips model, the maximum adsorption amounts of HA and FA at 298.5 K were 327.88 and 213.58 mg/g, respectively. The adsorption amount of HA was greater than that of FA, consistent with that of previous reports for adsorption of HSs to graphene oxides and carbon nanotubes [47]. This phenomenon was attributed to the abundance of aromatic rings and the polar nature of HA, which induced more robust π-π stacking interactions between HA and TCN-600 [34]. Compared to that of previously reported adsorbents (Table S6), the adsorbed amounts of both HA and FA by TCN-600 were greater. For example, palygorskite had a notable adsorption capacity for HA, with the maximum adsorption amount of 17 mg/g for HA at 298.5 K [48]. Thus, TCN-600 was highly effective for adsorbing HA. The thermodynamic parameters of HSs are summarized in Table S7. The very low $\Delta H^{0}$ values for adsorption of HSs to TCN-600 indicated that temperature was not a very important factor, suggesting a physisorption process. The negative $\Delta G^{0}$ values revealed that the adsorption progress was spontaneous.

In order to investigate the practical application of TCN, we studied the adsorption of HA and FA at low concentration (for simulating real surface waters) and adsorption of a landfill leachate concentrate on TCN-600 (Figures S7 and S8). It was found that the removal rates of HA and FA at a concentrate of 5~20 mg/L were above 90% and 60%, respectively, indicating that HSs can be removed effectively after adsorption using TCN. In addition, the removal rate measured as UV_254_ of landfill leachate concentrate reached 60.86%. EEM spectra showed that the fluorescent region of landfill leachate concentrate at Ex > 250 nm and Em > 380 nm was effectively eliminated, which was attributed to the humic-like components [49,50]. The above results indicated that HSs in surface waters and wastewaters can be effectively removed by adsorption using TCN.

### 3.7. Adsorption Mechanisms

**Figure 6:**
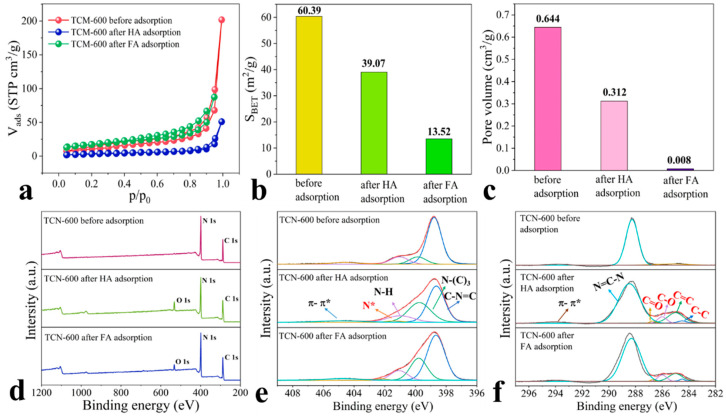
(**a**) The nitrogen adsorption–desorption isotherms, (**b**) specific surface area, (**c**) pore volume, (**d**) XPS spectra, (**e**) N 1s core region, and (**f**) C 1s core region before and after adsorption of HA and FA (C_HSs_ = 50 mg/L, pH = 3.0, C_TCN-600_ = 0.4 g/L t = 120 min, and I = 0.01 M).

The adsorption mechanisms for HSs on TCN were elucidated using nitrogen adsorption–desorption isotherms, XPS, and fluorescence EEM. As shown in Figure 6a, it was observed that after adsorption of HA and FA, and the hysteresis loops of isotherms shifted to higher relative pressures. This clearly indicated that HA and FA molecules diffused into the pore systems of TCN-600, which was consistent with the results of the intraparticle diffusion model. In addition, although the adsorption amounts of HA were greater than that of FA, the specific surface area and pore volume of TCN-600 both decreased more significantly after FA adsorption than HA (Figure 6b,c). This was because the average size of FA was less than that of HA, and as reported the average sizes of FA and HA molecules were 3.2 and 9.3 nm, respectively [51]. In this regard, more FA molecules could diffuse into the pore systems of TCN-600 than HA molecules. The similar diffusion mechanism was reported by Ateia et al. [52] for the adsorption of HA on carbon nanotubes.

As shown in Figure 6d and Table S8, after HA and FA adsorption, a new O 1s peak appeared at approximately 532.0 eV in the XPS spectra of TCN-600, indicating that the molecules of HSs were adsorbed on the surface of TCN-600. The N 1s and C 1s regions of TCN-600 after HSs adsorption were also decomposed. As shown in Figure 6e, a new N 1s peak appeared at 402.2 eV, which may be attributed to protonated or polarized nitrogen (N*) in the -NH_2_ and -NH- groups [22]. As reported in a previous study, amino groups have superior adhesion affinity towards HA [53]. As a result, the protonation and deprotonation of amine groups on the surface of TCN-600 played a critical role in HSs adsorption via electrostatic interactions (as discussed above). Collectively, we can conclude that electrostatic interactions were a major mechanism for HSs adsorption. As shown in Figure 6f, after HSs adsorption, four new peaks appeared in the C 1s region of TCN-600. The new peaks at 284.5 and 285.0 eV were attributed to carbonyl C=O and ether carbon C-O bonds, respectively, which may be the oxygen groups of HSs. The percentage of surface oxygen of TCN-600 increased from 1.41 to 7.90% and 4.66% after the adsorption of HA and FA, respectively (Table S2). The increases in the surface oxygen content after adsorption were consistent with the above discussions. The new peaks at 286.0 and 286.7 eV were attributed to aliphatic C-C and aromatic C=C bonds, respectively, which may be the carbon atom of HSs or to the combination with the carbon atom of TCN-600 [51]. In addition, HSs contain large quantities of carboxylic and phenolic moieties, which could act as hydrogen-bond donors [54]. The amino groups of TCN-600 could bind with the carboxylic and phenolic moieties of HSs via hydrogen bonding [55].

**Figure 7:**
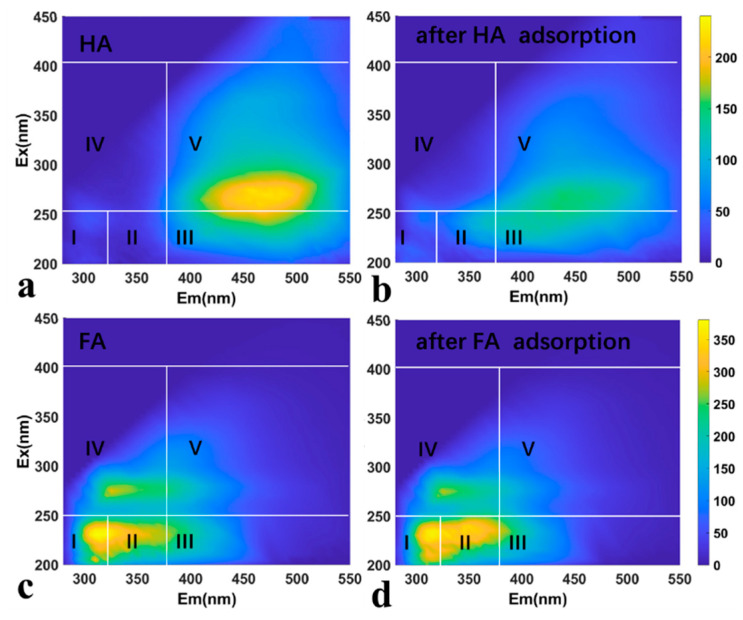
The EEM of HA and FA before (**a**,**c**) and after (**b**,**d**) adsorption by TCN-600 (C_HSs_ = 50 mg/L, pH = 3.0, t = 120 min, and I = 0.01 M).

In addition, HSs contain large quantities of aromatic rings, which promote π-π interactions with adsorbents containing aromatic rings. As shown in Figure 7, EEM was used to characterize HSs before and after adsorption. As previously reported [56], the fluorescence of HSs included five regions. Among them, FA-like components at Ex/Em = 200–250/380–550 nm and HA-like substances at Ex/Em = 250–400/380–550 nm. Thus, it was clear that HA-like and FA-like components were preferentially adsorbed at higher Ex/Em wavelengths. In particular, the II and V regions in the HA EEM showed decreased fluorescence intensity after adsorption; however, decreases in the II regions in the FA EEM were not clear. In general, the components in these regions have great aromaticity [57]. Thus, such a phenomenon can be explained by π-π interactions, indicating the presence of π-π interactions in HSs adsorption on TCN-600. Liu et al. [58] reported a similar finding in previous study. To sum up, adsorption of HSs to TCN-600 was mainly mediated by electrostatic interactions, hydrogen bonding, and π-π interactions. Particularly, the adsorption of HA was also affected by its variable conformation.

## 4. Conclusions

In this study, the synthesis of TCN using thermal oxidation was investigated, and the adsorption of HA and FA on TCN was evaluated. After thermal oxidation, the textural properties of TCN were remarkably affected, particularly in specific surface areas and pore size. As thermal oxidation temperature increased, the specific surface area of TCN at 600 °C (TCN-600) was 60.39 m^2^/g, much greater than that of bulk g-C_3_N_4_, enhancing the adsorption performance for HSs. The adsorption capacity of TCN-600 for HA was 145.35 mg/g, two times greater than that of bulk g-C_3_N_4_. The kinetics data were fitted well with the PSO and Elovich models, indicating HSs adsorption is related to chemisorption. Based on the Sips model, the maximum adsorption amounts of HA and FA at 298.5 K were quite substantial, calculated to be 327.88 and 213.58 mg/g, respectively. The adsorption capacity of TCN-600 was higher than that of most previously reported adsorbents. Moreover, TCN-600 could effectively remove HSs with low concentration. According to the intraparticle diffusion model, boundary layer diffusion and interior adsorption were involved in HSs adsorption. The adsorption of HSs was greatly affected by pH, alkali metals (Na^+^ and K^+^), and alkali earth metals (Ca^2+^ and Mg^2+^), which was due to electrostatic interactions and hydrogen bonding. HA-like and FA-like components were preferentially adsorbed at higher Ex/Em wavelengths, indicating the presence of π-π interactions. In particular, the adsorption of HA was also affected by the pH-dependent conformation. The findings are valuable for designing of HSs-target adsorbents and elimination of HSs from aqueous solutions.

## Data Availability

The data is confidential.

## Supplementary Materials

The following supporting information can be downloaded at: https://www.mdpi.com/article/10.3390/toxics11040369/s1, Figure S1: The experimental instrument used in this study; Figure S2: The adsorption capacities and removal rate (%) of HA by the bulk g-C3N4 and TCNs; Figure S3: The adsorption kinetics of HA on the bulk g-C3N4; Figure S4: The effect of pH for adsorption of HSs on TCN-600; Figure S5: The zeta potential of TCN-600 as a function of pH; Figure S6: The particle size distributions of HA and FA as a function of pH; Figure S7: The removal rate of HA and FA at low initial concentration; Figure S8: The removal rates measured as UV254 of landfill leachate concentrate on the TCN-600; Table S1: The detailed experimental parameters adopted in this study; Table S2: The relative content of surface elements (%) of XPS spectra peaks of the bulk g-C3N4 and TCNs before and after adsorption of HSs; Table S3: The relative contents (%) of peaks in the N 1s and C 1s core region of the bulk g-C3N4 and TCNs; Table S4: Kinetics parameters for adsorption of HSs on the bulk g-C3N4 and TCNs; Table S5: The parameters of adsorption isotherms for adsorption of HSs on TCN-600; Table S6: A comparison for adsorption of HSs on various adsorbents; Table S7: Thermodynamic parameters for adsorption of HSs on TCN-600; Table S8: The relative contents (%) of peaks in the N 1s and C 1s core region of TCN-600 before and after adsorption of HSs. References [59,60,61,62,63,64,65,66,67] are cited in the Supplementary Materials.
