# Supplementary material for: The Adsorption Behaviors and Mechanisms of Humic Substances by Thermally Oxidized Graphitic Carbon Nitride
Source: Toxics. 2023 Apr 12;11(4):369. doi: 10.3390/toxics11040369 (PMC10142187; doi:10.3390/toxics11040369)
Supplement: Supplementary file 1 [file toxics-11-00369-s001.zip › toxics-2318926-supplementary.pdf]

toxics

MDPI

Article

The Adsorption Behaviors and Mechanisms of Humic Substances by Thermally Oxidized Graphitic Carbon Nitride

Hongxin Li [1,2], Jianlong Wang [1], Dongbei Yue [2,*], Jianchao Wang [3], Chu Tang [2] and Lingyue Zhang [4]

[1] School of Environment and Energy Engineering, Beijing University of Civil Engineering and Architecture, Beijing, 100044, China
[2] School of Environment, Tsinghua University, Beijing 100084, China
[3] School of Chemical and Environmental Engineering, China University of Mining and Technology (Beijing), Beijing, 100083, China
[4] School of Department of Civil Engineering, The University of Hong Kong, Pokfulam, Hong Kong, SAR 999077, China
* Correspondence: author: Dongbei Yue (E-mail: yuedb@tsinghua.edu.cn; phone/Fax number: +86 10–62771931)

Supporting Information

Figures:

Figure S1 The experimental instrument used in this study.
Figure S2 The adsorption capacities and removal rate (%) of HA by the bulk g-C_3N_4 and TCNs.
Figure S3 The adsorption kinetics of HA on the bulk g-C_3N_4.
Figure S4 The effect of pH for adsorption of HSs on TCN-600.
Figure S5 The zeta potential of TCN-600 as a function of pH.
Figure S6 The particle size distributions of HA and FA as a function of pH.
Figure S7 The removal rate of HA and FA at low initial concentration.
Figure S8 The removal rates measured as UV_{254} of landfill leachate concentrate on the TCN-600.

Tables:

Table S1 The detailed experimental parameters adopted in this study.
Table S2 The relative content of surface elements (%) of XPS spectra peaks of the bulk g-C_3N_4 and TCNs before and after adsorption of HSs.
Table S3 The relative contents (%) of peaks in the N 1s and C 1s core region of the bulk g-C_3N_4 and TCNs.
Table S4 Kinetics parameters for adsorption of HSs on the bulk g-C_3N_4 and TCNs.
Table S5 The parameters of adsorption isotherms for adsorption of HSs on TCN-600.
Table S6 A comparison for adsorption of HSs on various adsorbents.
Table S7 Thermodynamic parameters for adsorption of HSs on TCN-600.
Table S8 The relative contents (%) of peaks in the N 1s and C 1s core region of TCN-600 before and after adsorption of HSs.

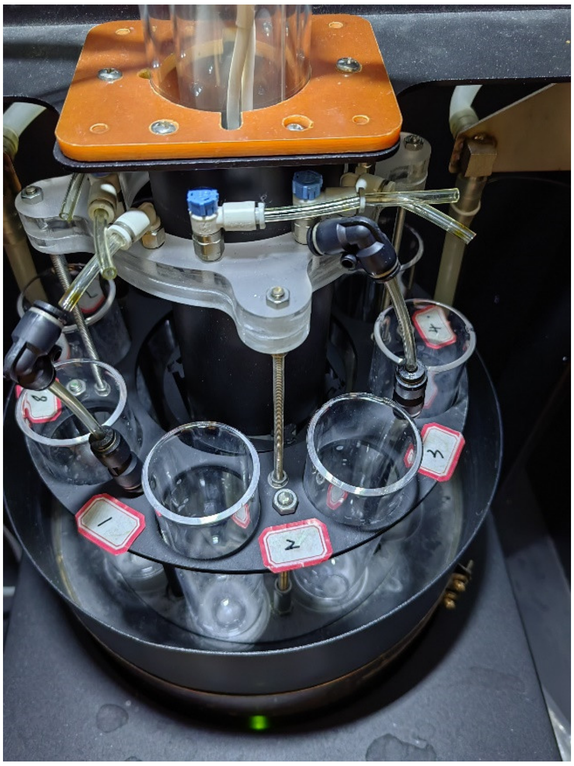

Figure S1. The experimental instrument used in this study.

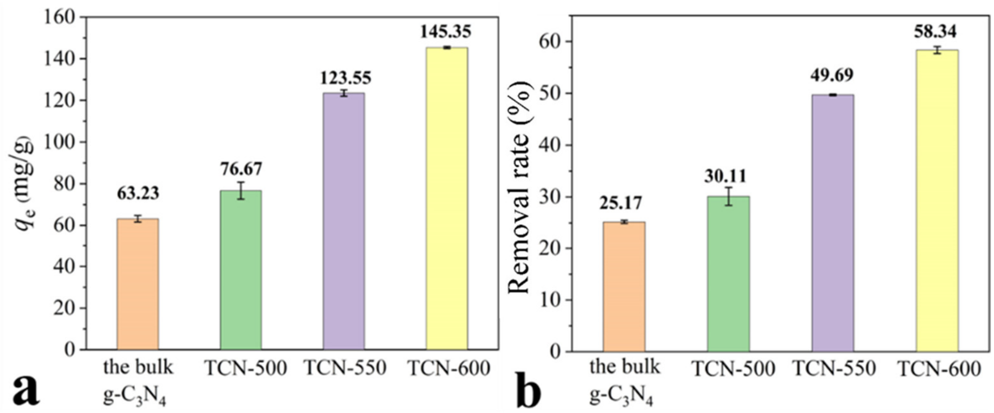

Figure S2. The adsorption capacities (a) and removal rate (%) (b) of HA by the bulk g-C_3N_4 and TCNs (C_{HA} = 100 mg/L, pH = 3.0, T = 298 K, the adsorbents C = 0.4 g/L, t = 240 min, and I = 0.01 M).

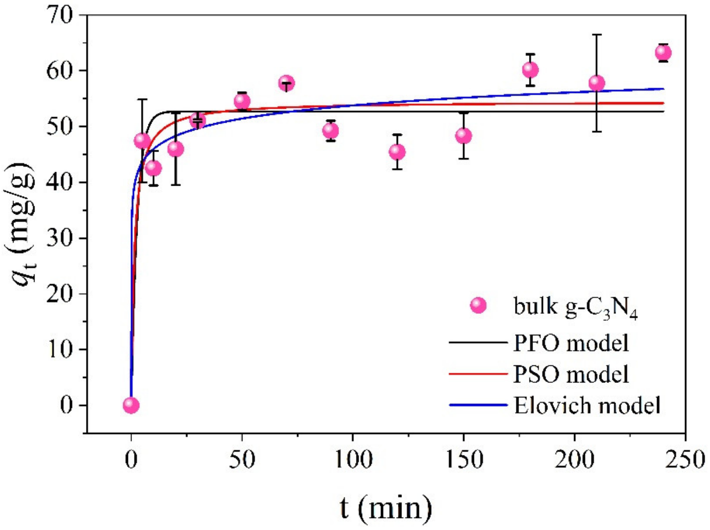

Figure S3. The adsorption kinetics of HA on the bulk g-C_3N_4 (C_{HSs} = 100 mg/L, pH = 3.0, T = 298.15 K, C = 0.4 g/L, and I = 0.01 M).

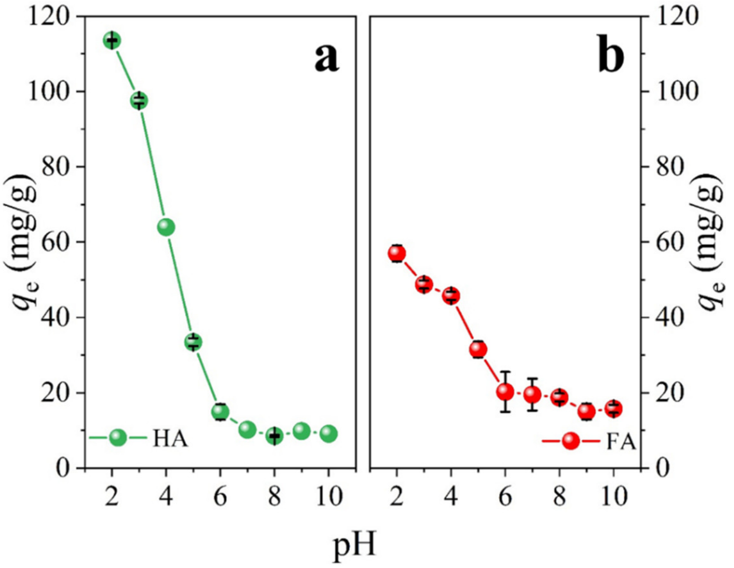

Figure S4. The effect of pH on the adsorption of HA **(a)** and FA **(b)** on TCN-600 (C_{HSs} = 50 mg/L, pH = 2.0–10.0, T = 298.15 K, $C_{TCN-600}$ = 0.4 g/L, and I = 0.01 M).

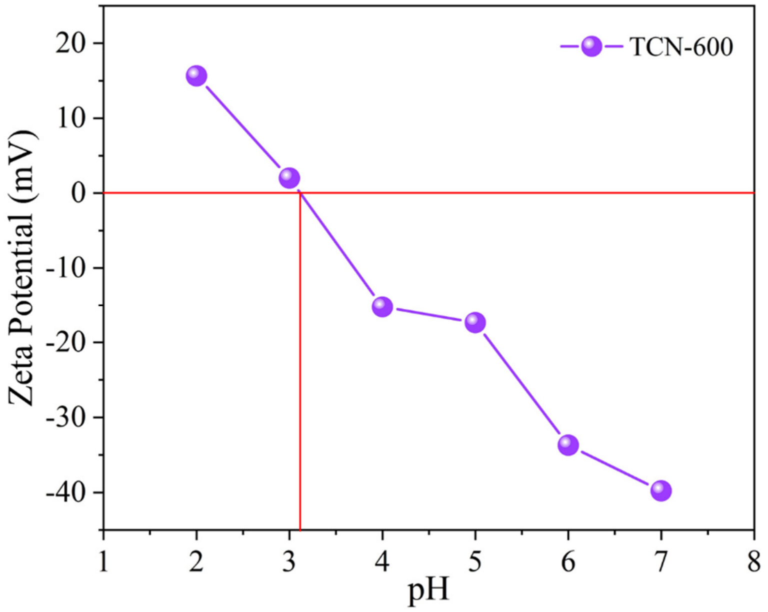

Figure S5. The zeta potential of TCN-600 as a function of pH (TCN-600 = 1 g/L).

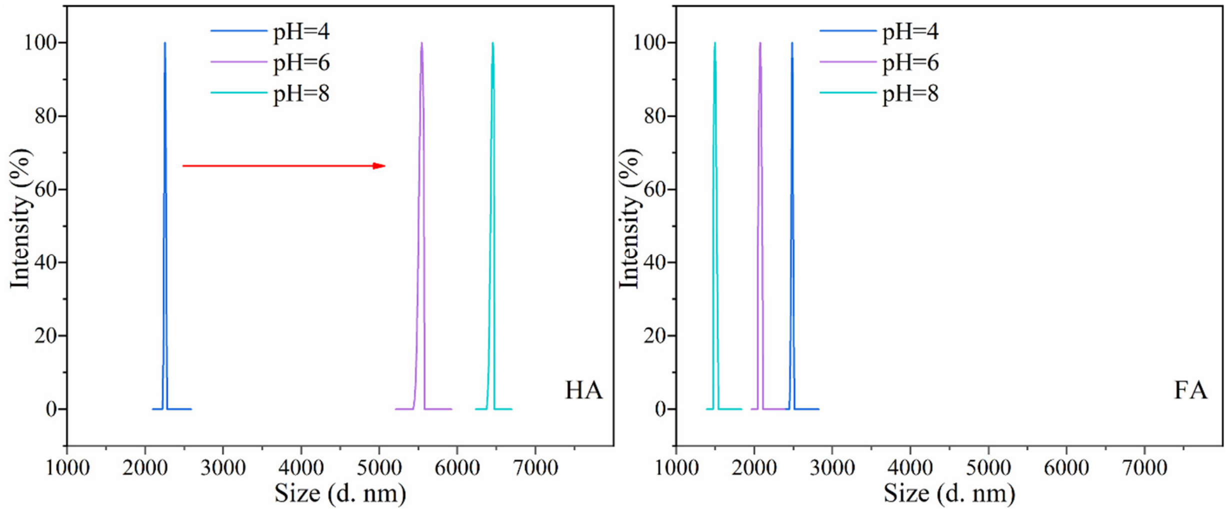

Figure S6. The particle size distributions of HA and FA as a function of pH.

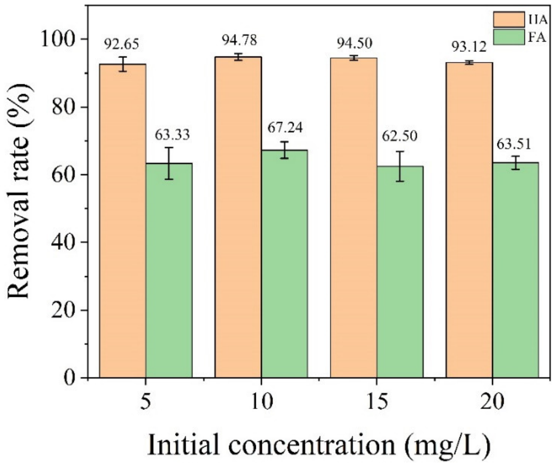

Figure S7. The removal rate of HA and FA at low initial concentration (C_{HSs} = 5–20 mg/L, pH = 3.0, T = 298.15 K, $C_{TCN-600}$ = 0.4 g/L, and I = 0.01 M).

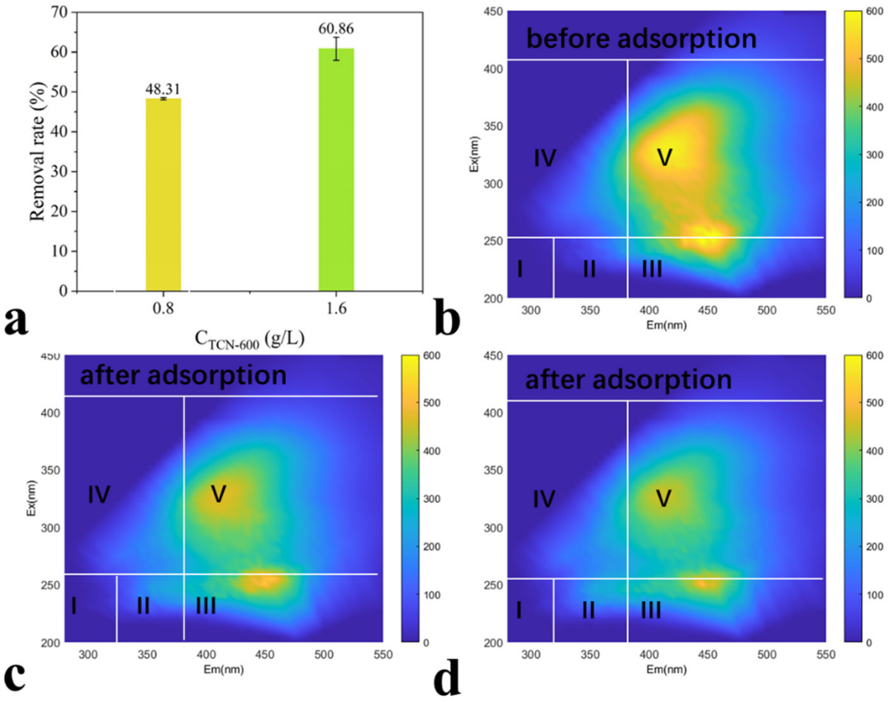

Figure S8. The removal rates measured as UV_{254} of landfill leachate concentrate on the TCN-600 (a), and EEM of landfill leachate concentrate before (b) and after adsorption (c) (TCN-600 = 0.8 g/L) and (d) (TCN-600 = 1.6 g/L) （C_{HSs} = 40 mgC/L, pH = 3.0, T = 298.15 K, t=60 min）.

Table S1. The detailed experimental conditions adopted in this study.

Procedure	t (min)	pH	C_{HSs} (mg/L)	I (M)	T (K)	C (g/L)	K^+ (M)	Ca^{2+} (mM)	Mg^{2+} (mM)
Adsorbent Capacities	240	3	100	0.01	298.15	0.4	0	0	0
Kinetics	0–240	3	100/50	0.01	298.15	0.4	0	0	0
Intraparticle Diffusion	0–240	3	100/50	0.01	298.15	0.4	0	0	0
Adsorption Isotherms	120	3	25–200	0.01	298.15<br>308.15<br>318.15	0.4	0	0	0
Low Initial Concentration Adsorption	120	3	5–20	0.01	298.15	0.4	0	0	0
pH	120	2–10	50	0.01	298.15	0.4	0	0	0
Na^+	120	3	50	0–0.08	298.15	0.4	0	0	0
K^+	120	3	50	0.01	298.15	0.4	0–0.07	0	0
Ca^{2+}	120	3	50	0.01	298.15	0.4	0	0–1.05	0
Mg^{2+}	120	3	50	0.01	298.15	0.4	0	0	0–1.05
Adsorption Landfill Leachate	60	3	40 (mgC/L)	0	298.15	0.4	0	0	0
Nitrogen Adsorption/Desorption Isotherms	120	3	20	0.01	298.15	0.4	0	0	0
XPS Analysis	120	3	20	0.01	298.15	0.4	0	0	0

Contact time (t, min); HSs concentration (C_{HSs}, mg/L); ionic strength (I, M); temperature (T, K); the bulk $g-C_3N_4$ or TCN concentration (C, g).

Table S2. The relative content of surface elements (%) of XPS spectra peaks of the bulk g-C_3N_4 and TCNs before and after adsorption of HSs.

	CNs	C Content (%)	N Content (%)	O Content (%)	C/N (ato. %)
Before Adsorption	The bulk g-C_3N_4	41.69	56.36	1.95	0.740
	TCN-500	41.83	56.28	1.89	0.743
	TCN-550	42.02	56.00	1.98	0.750
	TCN-600	40.94	57.65	1.41	0.710
After Adsorption	TCN-600 (after HA adsorption)	45.49	46.61	7.90	0.976
	TCN-600 (after FA adsorption)	44.34	51.00	4.66	0.869

Table S3. The relative contents (%) of peaks in the N 1s and C 1s core region of the bulk g-C_3N_4 and TCNs.

The Types of Core Region	Assignments	Position (eV)	Relative Contents (%)			
			The Bulk g-C_3N_4	TCN-500	TCN-550	TCN-600
**N 1s**	C-N=C	398.8	70.25	68.07	70.94	69.74
	N-$(C)_3$	399.6	14.19	14.11	13.49	11.40
	N–H	400.9	11.86	13.90	11.68	12.40
	π-π*	404.5	3.70	3.92	3.90	6.45
**C 1s**	CO_3^{2-}	284.8	10.01	11.14	11.79	5.90
	N=C-N	288.3	87.49	86.36	86.01	91.53
	π-π*	293.8	2.49	2.50	2.20	2.56

Table S4. Kinetics parameters for adsorption of HSs on the bulk g-C_3N_4 and TCNs.

	HSs	Pseudo-First-Order-Model			Pseudo-Second-Order-Model			Elovich Model			$q_{e(e)}$ (mg/g)
		$q_{e(c)}$ (mg/g)	k_1 (1/min)	R_1^2	$q_{e(c)}$ (mg/g)	k_2 (g/mg·min)	R_2^2	β (g/mg)	α (mg/g·min)	R_3^2	
The Bulk g-C_3N_4	HA	52.718	0.852	0.838	54.514	0.218	0.881	0.294	255339.702	0.906	63.228
TCN-500	HA	70.440	0.157	0.877	74.451	0.003	0.934	0.127	599.475	0.966	76.673
TCN-550	HA	103.301	0.146	0.796	111.879	0.002	0.893	0.073	308.622	0.971	123.816
TCN-600	HA	119.711	0.164	0.826	128.583	0.002	0.908	0.068	665.766	0.974	145.349
	FA	44.798	0.344	0.971	45.982	0.017	0.989	0.461	2.410	0.991	46.832

Table S5. The parameters of adsorption isotherms for adsorption of HSs on TCN-600.

Isotherm models		HA-Temperature (K)			FA-Temperature (K)		
		298.15	308.15	318.15	298.15	308.15	318.15
Freundlich Isotherm Model	$1/n$	0.394	0.365	0.439	0.510	0.482	0.528
	k_f ((mg /g) (L/mg)1/n)	28.317	36.856	37.801	8.396	9.794	8.480
	R^2	0.980	0.965	0.980	0.979	0.960	0.983
Langmuir Isotherm Model	b	0.034	0.052	0.031	0.015	0.018	0.014
	q_m (mg/g)	221.353	226.778	358.739	149.879	144.444	170.172
	R^2	0.887	0.884	0.938	0.981	0.963	0.987

Sips Isotherm Model	q_m (mg/g)	327.879	344.771	399.766	213.575	224.371	235.362
	k_s (L/mg)	0.012	0.015	0.025	0.006	0.006	0.007
	$1/n$	0.667	0.621	0.877	0.769	0.714	0.794
	R^2	0.947	0.943	0.951	0.985	0.966	0.989

Table S6. A comparison for adsorption of HSs on various adsorbents.

Adsorbent	q_{m-HA} (mg/g)	q_{m-FA} (mg/g)	References
Bentonite Nanoparticles	58.21	\	1
Montmorillonite Nanoparticles	48.20	\	
Chitosan-H_2SO_4 Beads	377.40	\	2
Acid-Activated Greek Bentonite	10.75	\	3
Layered Double Hydroxides/Hollow Carbon Microsphere Composites	300.46	\	4
Powder Activated Carbon (PAC)	70.00	\	5
SBA-15	8.50	\	6
Algerian Bentonite	54.80	\	7
Fly Ash	36.00	\	8
Magnetic Chitosan	32.6	\	9
Cellulose Acetate/Chitosan Nanofiber	238.10	\	10
Amine-Functionalized Mesoporous Silica	\	39.5	11
Magnetic Graphene Oxide	98.82	72.4	12
This Study	331.31	185.93	

Table S7. Thermodynamic parameters for adsorption of HSs on TCN-600.

HSs	Temperature (K)	ΔG^0 (kJ/mol)	ΔH^0 (kJ/mol)	ΔS^0 (J/mol K)
HA	298	-4.062	-15.708	-39.017
	308	-4.296		-36.992
	318	-3.923		-37.003
FA	298	-4.331	-4.652	-1.074
	308	-4.282		-1.199
	318	-4.257		-1.239

Table S8. The relative contents (%) of peaks in the N 1s and C 1s core region of TCN-600 before and after adsorption of HSs.

The Types of Core Region	Assignments	Position (eV)	Relative Contents (%)		
			TCN-600 Before Adsorption	TCN-600 After HA Adsorption	TCN-600 After FA Adsorption
**N 1s**	C-N=C	398.8	69.74	47.46	56.31
	N-$(C)_3$	399.6	11.40	33.87	25.98
	N–H	400.9	12.40	12.65	11.20
	N*	402.2	\	1.44	1.15
	π-π*	404.5	6.45	4.58	5.36
**C 1s**	C-C	284.5	\	5.06	1.93
	CO_3^{2-}	284.8	5.90	\	
	C=C	285.0	\	13.11	11.71
	C-O	286.0	\	4.69	5.64

C=O	286.7	\	1.18	0.83
N=C-N	288.3	91.53	73.43	78.14
π-π*	293.8	2.56	2.53	1.75

References

1. Derakhshani, E.; Naghizadeh, A., Optimization of humic acid removal by adsorption onto bentonite and montmorillonite nanoparticles. *J. Mol. Liq.* **2018,** *259*, 76–81.
2. Ngah, W. S. W.; Fatinathan, S.; Yosop, N. A., Isotherm and kinetic studies on the adsorption of humic acid onto chitosan-H_2SO_4 beads. *Desalination* **2011,** *272* (1–3), 293–300.
3. Doulia, D.; Leodopoulos, C.; Gimouhopoulos, K.; Rigas, F., Adsorption of humic acid on acid-activated Greek bentonite. *J. Colloid Interface Sci.* **2009,** *340* (2), 131–141.
4. Huang, S. Y.; Song, S.; Zhang, R.; Wen, T.; Wang, X. X.; Yu, S. J.; Song, W. C.; Hayat, T.; Alsaedi, A.; Wang, X. K., Construction of Layered Double Hydroxides/Hollow Carbon Microsphere Composites and Its Applications for Mutual Removal of Pb(II) and Humic Acid from Aqueous Solutions. *ACS Sustain. Chem. Eng.* **2017,** *5* (12), 11268–11279.
5. Yang, K.; Fox, J. T., Adsorption of Humic Acid by Acid-Modified Granular Activated Carbon and Powder Activated Carbon. *J. Environ. Eng.-ASCE* **2018,** *144* (10), 10.
6. Tao, Q.; Xu, Z. Y.; Wang, J. H.; Liu, F. L.; Wan, H. Q.; Zheng, S. R., Adsorption of humic acid to aminopropyl functionalized SBA-15. *Microporous Mesoporous Mater.* **2010,** *131* (1–3), 177–185.
7. Wang, Q. Z.; Chen, X. G.; Liu, N.; Wang, S. X.; Liu, C. S.; Meng, X. H.; Liu, C. G., Protonation constants of chitosan with different molecular weight and degree of deacetylation. *Carbohydr. Polym.* **2006,** *65* (2), 194–201.
8. Wang, S. B.; Terdkiatburana, T.; Tade, M. O., Single and co-adsorption of heavy metals and humic acid on fly ash. *Sep. Purif. Technol.* **2008,** *58* (3), 353–358.
9. Dong, C. L.; Chen, W.; Liu, C., Preparation of novel magnetic chitosan nanoparticle and its application for removal of humic acid from aqueous solution. *Appl. Surf. Sci.* **2014,** *292*, 1067–1076.
10. Zhang, Y. R.; Wang, F.; Wang, Y. X., Electrospun Cellulose Acetate/Chitosan Fibers for Humic Acid Removal: Construction Guided by Intermolecular Interaction Study. *ACS Appl. Polym. Mater.* **2021,** *3* (10), 5022–5029.
11. Jayalath, S.; Larsen, S. C.; Grassian, V. H., Surface adsorption of Nordic aquatic fulvic acid on amine-functionalized and non-functionalized mesoporous silica nanoparticles. *Environ. Sci.-Nano* **2018,** *5* (9), 2162–2171.
12. Zhang, J.; Gong, J. L.; Zenga, G. M.; Ou, X. M.; Jiang, Y.; Chang, Y. N.; Guo, M.; Zhang, C.; Liu, H. Y., Simultaneous removal of humic acid/fulvic acid and lead from landfill leachate using magnetic graphene oxide. *Appl. Surf. Sci.* **2016,** *370*, 335–350.

Disclaimer/Publisher's Note: The statements, opinions and data contained in all publications are solely those of the individual author(s) and contributor(s) and not of MDPI and/or the editor(s). MDPI and/or the editor(s) disclaim responsibility for any injury to people or property resulting from any ideas, methods, instructions or products referred to in the content.
